# Interferon-gamma release assay determined tuberculosis infection: prevalence and risk factors among community-dwelling elderly in Huzhou, a low-endemic region of China

**DOI:** 10.1186/s12877-026-08032-2

**Published:** 2026-08-17

**Authors:** Feilin Ren, Jie Tian, Jian Zhang, Meihua Jin, Bin Chen, Zhongrong Yang, Sichao Zhang, Peng Zhang, Wei Wang, Weibing Wang, Jianyong Shen

**Affiliations:** 1 Huzhou Center for Disease Control and Prevention, Huzhou, 313000 Zhejiang China; 2 Shanghai Institute of Infectious Disease and Biosecurity, School of Public Health, Fudan University, Shanghai, China; 3 Department of Epidemiology, School of Public Health, Fudan University, Shanghai, China; 4 Changxing County Center for Disease Control and Prevention, Changxing, Huzhou, 313100 Zhejiang China; 5 Zhejiang Provincial Center for Disease Control and Prevention, Hangzhou, 310051 Zhejiang China; 6 School of Public Health, Fudan University, Shanghai, China

**Keywords:** Tuberculosis infection, Interferon-gamma release assays (IGRA), Fasting blood glucose (FBG), Risk factors

## Abstract

**Background:**

Tuberculosis (TB) remains the leading cause of death from a single infectious agent worldwide. This study was conducted in Huzhou City, a low-TB-incidence areas, to investigate the prevalence of tuberculosis infection (TBI) and its associated risk factors among rural elderly population. The findings aim to inform TB elimination strategies in similar low-incidence settings.

**Methods:**

A cross-sectional study was conducted from May to August 2023 among 1,258 rural residents aged ≥ 65 years in Changxing County, Huzhou City. Data were collected through health examinations, structured questionnaires, and interferon-gamma release assay (IGRA) testing using the QuantiFERON-TB Gold kit (ELISA). Chi-square tests, t-tests, and Fisher’s exact tests were used to compare TBI prevalence across subgroups. Variables with *P* < 0.05 in univariate analyses were entered into a multivariate logistic regression model. A multi-model strategy was applied to evaluate the association between abnormal imaging findings and TBI. Restricted cubic splines (RCS) were used to examine the dose-response relationship between blood glucose levels and TBI.

**Results:**

Among the 1,032 enrolled permanent residents aged ≥ 65 years, the IGRA-positive was 9.79% (95% CI: 8.12%–11.75%). Significant differences were observed between IGRA-positive and IGRA-negative individuals in terms of TB history, abnormal chest radiograph findings, sex, immunosuppressive diseases, and smoking status (all *P* < 0.05). Multivariate analysis identified TB history, abnormal chest radiograph findings, and immunosuppressive diseases as independent risk factors for TBI. Individuals with a prior TB history had 8.49-fold higher odds of TBI (95% CI: 2.01–35.81) versus those without, while abnormal chest radiographs were linked to 1.64-fold higher odds of TBI (95% CI: 1.07–2.50). The multi-model analysis further confirmed that abnormal chest radiograph findings remained a robust predictor of TBI. Additionally, restricted cubic spline analysis revealed a negative dose-response association between blood glucose levels and the risk of TBI.

**Conclusions:**

In low-incidence areas, individuals with a prior TB history show significantly elevated IGRA positivity. The link between TBI and abnormal chest imaging suggests a practical role for radiography in screening. The inverse association of fasting blood glucose (FBG) with IGRA positivity introduces a new immunologic insight for aging populations that requires further exploration. Collectively, these findings deliver actionable local evidence to inform targeted screening strategies for the elderly.

**Supplementary Information:**

The online version contains supplementary material available at https://doi.org/10.1186/s12877-026-08032-2.

## Introduction

Tuberculosis (TB) remains the leading cause of death from a single infectious agent globally. An estimated global total of 10.7 million people fell ill with TB in 2024, with an estimated 1.08 million deaths among HIV-negative people [1]. A pivotal element sustaining this epidemic is tuberculosis infection (TBI), a condition characterized by a persistent immune response to *Mycobacterium* tuberculosis (*M.tb*) antigens in the absence of clinically active disease. Current models indicate that roughly a quarter of the global population lives with TBI, representing a vast reservoir for future TB cases. Individuals with TBI are typically asymptomatic and non-infectious but face a lifelong 5–10% cumulative risk of progressing to active TB, with approximately half of these reactivations occurring within the first two years post-infection [2]. The World Health Organization (WHO) endorses the use of Interferon-Gamma Release Assays (IGRA) for TBI detection due to their high specificity,, particularly in BCG-vaccinated populations [3, 4].

Risk factors for TBI can be broadly classified into two categories: environmental exposure to *M.tb* and host-related immune status. High-risk environments typically involve close and prolonged contact with infectious TB cases, such as household exposure [5, 6], as well as congregate settings like prisons, detention centers, and long-term care facilities. Healthcare workers who are frequently in contact with undiagnosed or active TB patients also constitute a recognized high-risk group [7]. From the host perspective, immunosuppression significantly increases susceptibility to TBI, even with limited bacterial exposure [8, 9]. This includes conditions such as HIV infection, which leads to progressive depletion of CD4⁺ T-cells, iatrogenic immunosuppression in transplant recipients, and other comorbidities associated with immunity. These include silicosis (involving impaired pulmonary macrophage function), poorly controlled diabetes mellitus (linked to dysregulated neutrophil and T-cell responses and increased risk of progression to active TB) [10], advanced malignancies, and chemotherapy—all of which contribute to elevated TBI risk.

The interaction between diabetes mellitus (DM) and TBI is of particular concern. Individuals with DM are not only at a higher risk of acquiring *M.tb* infection (i.e., developing TBI) but also face an increased likelihood of progression from TBI to active TB. Previous study reveals that the risk of TBI was found to be a 60% increase in DM patients, compared with non-DM patients [11, 12]. Another meta-analysis of 13 observational studies found that the risk of TBI was significantly higher in individuals with DM compared to those without (pooled odds ratio [OR] = 1.18). This association was more pronounced in individuals under 60 years of age and in those with poor glycemic control (HbA1c > 7%) [13]. Mechanistically, hyperglycemia and insulin resistance impair key functions of innate and cellular immunity, particularly of macrophages and T-cells, thereby facilitating initial *M.tb* infection and subsequent reactivation.

Huzhou City in Zhejiang Province is a low-TB-incidence area, with an annual reported incidence of 28.36 per 100,000 population in 2023 [14]. Since 2022, in alignment with the national “End TB” strategy, Huzhou has pioneered a large-scale, community-based screening initiative utilizing IGRA. While previous studies [15–21] in China have investigated TBI prevalence in high-risk groups such as school contacts, adolescents, coal workers, village doctors, and prisoners, a significant gap remains regarding large-scale surveys among general community-dwelling healthy elderly populations. Furthermore, current national TBI burden rely on modeling estimates, which are inherently constrained by the quality and availability of input data, as well as the specific assumptions of each model [22]. This study bridges this gap by presenting the first population-based TBI prevalence data derived from this extensive screening effort and elucidates the associated risk factors. Our findings provide novel empirical data that are critical for shaping evidence-based public health interventions and optimizing future screening strategies in low-endemic settings.

## Methods

### Study design and participants

A cross-sectional study was conducted from May to August 2023 in Heping Town, Changxing County, Huzhou City, Zhejiang Province, China. Although Huzhou is a low tuberculosis-endemic region, local TB incidence shows obvious spatial heterogeneity. We applied a stepwise stratified cluster sampling strategy based on relative local TB incidence. According to the 2023 surveillance data, Changxing County with a relatively higher TB incidence was selected. We further selected Heping Town with the second-highest average TB incidence (2020–2023) among 15 townships, and finally enrolled the rural residential area with the highest local TB incidence as the study site.The study enrolled residents aged 65 years or older who had been living in the village for at least six months prior to the survey. Individuals with active tuberculosis, cognitive impairment, or limited legal capacity were excluded. The sample size was calculated using the formula for cross-sectional studies, with an assumed prevalence of IGRA-positive individuals estimated at 34.9% based on previous studies and literature reports [23, 24]. With a 95% confidence level and a margin of error of 5%, a design effect (Deff) of 1.5 was applied to account for clustering in the rural elderly population. Additionally, 10% was added to compensate for potential invalid responses. The calculated minimum sample size was 910 participants, and a total of 1032 individuals were ultimately included. In 2023, the village consisted of 23 natural residential areas, with a total of 1,258 elderly residents aged 65 years and above. The final analytical sample consisted of 1,032 participants who completed both the questionnaire and IGRA testing, representing a response rate 82.03%. In this study, TBI was defined as a positive IGRA result in the absence of clinical, bacteriological, or radiographic evidence of active TB.

### Data source and data collection

Basic demographic characteristics, outdoor activities and social behaviors of the subjects were collected from case questionnaires via a self-designed survey [21]. Relevant laboratory indicators (blood routine, blood glucose, etc.) were extracted from outpatient examination records. History of TB and immunosuppressive diseases was first obtained by questionnaire, then verified and confirmed in the Zhejiang Provincial Urban and Rural Resident Health Examination Information System registry. Data on outdoor activities were collected via on-site questionnaires. To identify immunosuppressive diseases, participants were first interviewed regarding relevant illnesses; self-reported conditions were subsequently cross-verified with their health records for confirmation. Face-to-face questionnaire interviews were carried out at the village clinic. TB infection laboratory testing results were derived from IGRA assay reports, with the QuantiFERON-TB Gold kit (ELISA) procured from QIAGEN Enterprise Management (Shanghai) Co., Ltd. Imaging evaluation findings were sourced from diagnostic reports issued by the Radiology Department of Heping Town Community Health Center.

The following clinical variables were recorded:1) Demographic characteristics: sex, age, place of residence; 2) Behavioral factors: history of smoking, history of alcohol use; exercise frequency, smoking status, alcohol drinking status, frequency of regular window ventilation at home, and average daily total sleep duration, The main activity venues of the participants per week (e.g., entertainment venues, cultural auditoriums, street-side shops and general stores, vegetable markets, etc.) 3) Medical history: history of diabetes diagnosis, history of seven immunosuppressive diseases including systemic lupus erythematosus (refer to variable definition table); 4) Physical measures: height, weight, body mass index (BMI); 5) Laboratory indicators: various blood cell counts, blood glucose, serum creatinine, among others; 6) Imaging results: chest X-ray findings; 7) Immunosuppressive diseases were defined as the presence of at least one of the following: diabetes mellitus, hyperthyroidism, myasthenia gravis, systemic lupus erythematosus (SLE), rheumatoid arthritis (RA), ankylosing spondylitis (AS), and AIDS. 8) TB infection status: results of IGRA.

### Statistical analysis

Descriptive statistics were computed to summarize the study population. TBI prevalence was presented as the proportion of IGRA-positive individuals, with 95% confidence intervals (CIs). Categorical variables (e.g., sex, history of TB) were reported as frequencies and percentages. Continuous variables (e.g., age, BMI) were assessed for normality using the Kolmogorov–Smirnov test. Based on this assessment, normally distributed variables were expressed as means with standard deviations (SDs), while non-normally distributed variables were reported as medians with interquartile ranges (IQRs).

Bivariate Analysis were performed to evaluate the associations between TBI and potential risk factors were evaluated using chi-square tests for categorical variables or Fisher’s exact tests for sparse data (expected cell counts < 5). Differences in continuous variables were compared using the Student’s *t*-test for normal distributed data or the Mann–Whitney U test for non-normal distributed data.

Multivariate Analysis: Variables with *p* ≤ 0.05 in bivariate analysis were included in a multivariate logistic regression model to identify independent risk factors for TBI. Adjusted odds ratios (aORs) with 95% CIs were calculated. The enter method selection was used to refine the model. Meanwhile, a multi-model strategy was applied to systematically evaluate the association between abnormal imaging findings from health examinations and TBI. Additionally, restricted cubic splines (RCS) analysis (with knots set at the 10th, 50th, and 90th percentiles) was utilized to accurately characterize the dose-response relationship between blood glucose levels and TBI, avoiding potential bias induced by the linearity assumption.

Software and Significance: All statistical analyses were performed using R software (Version 4.5.1; R Foundation for Statistical Computing, Vienna, Austria). Statistical significance was defined as *P* ≤ 0.05 (two-tailed).

## Results

### Baseline characteristics of the study population

This study enrolled a total of 1,032 permanent residents aged 65 years or older from Huzhou City. Among them, 931 individuals (90.21%) tested negative for the IGRA, while 101 individuals (9.79%, 95% Confidence Interval: 8.12% − 11.75%) tested positive. As shown in Table 1, the cohort comprised 549 females (53.20%) and 483 males (46.80%), with a mean age of 72.83 ± 5.92 years and a mean body mass index (BMI) of 23.38 ± 3.29 kg/m². Comparative analysis of demographic characteristics between the IGRA-positive and IGRA-negative groups revealed no statistically significant differences in age or BMI (*P* > 0.05 for both). However, a significant disparity was observed in sex distribution. The IGRA positivity rate was significantly higher in males (12.22%) than in females. The distribution of the main differences between the two groups is shown in Table 1. Other variables examined showed no statistically significant associations and are reported in the Supplementary Materials.

**Table 1:** Characteristics of the community-based elderly population (≥ 65 years) of the participants

Variables	Number of participants (*n* = 1032)	IGRA Negative (*n* = 931)	IGRA Positive (*n* = 101)	IGRA Positive Rates (%)	*P*
Age, Mean ± SD	72.83 ± 5.92	72.85 ± 5.99	72.55 ± 5.31	-	0.629
Sex, n(%)					0.014
Female	549	507 (54.46)	42 (41.58)	7.65	
Male	483	424 (45.54)	59 (58.42)	12.22	
BMI, Mean ± SD	23.38 ± 3.29	23.42 ± 3.32	22.99 ± 2.99	-	0.221
BMI groups, n(%)					0.147
Normal	571	518 (55.64)	53 (52.48)	9.28	
Overweight	329	295 (31.69)	34 (33.66)	10.33	
Obese	82	77 (8.27)	5 (4.95)	6.10	
underweight	50	41 (4.40)	9 (8.91)	18.00	
DR diagnosis, n(%)					**0.015**
Normal	548	506 (54.35)	42 (41.58)	7.66	
Abnormal	484	425 (45.65)	59 (58.42)	12.19	
History of TB, n(%)					**0.004**
No	1024	927 (99.57)	97 (96.04)	9.47	
Yes	8	4 (0.43)	4 (3.96)	50.00	
Smoke, n(%)					**0.006**
No	806	738 (79.27)	68 (67.33)	8.44	
Yes	226	193 (20.73)	33 (32.67)	14.6	
Immunosuppressive diseases, n(%)					**0.011**
No	926	828 (88.94)	98 (97.03)	10.58	
Yes	106	103 (11.06)	3 (2.97)	2.83	

### Associations between risk factors and TBI

Variables with significant associations in the univariable analysis (sex, history of TB, abnormal chest radiograph findings, and presence of immunosuppressive diseases, all *P* < 0.05) were included in a multivariable logistic regression model. After adjusting for confounding factors, a history of TB, abnormal chest radiograph findings, and immunosuppressive diseases remained independently and significantly associated with TBI. Specifically, individuals with a previous TB history had 8.49 times higher odds of TB infection (95% CI: 2.01–35.81) compared to those without. Similarly, participants with abnormal chest radiographs had 1.64 times higher odds of TB infection (95% CI: 1.07–2.50) than those with normal findings (Table 2).

**Table 2:** Univariate and multivariable logistic regression analysis of factors associated with TB infection

Variables	Univariable Analysis					Multivariable Analysis				
	β	S.E	Z	*P*	OR (95%CI)	β	S.E	Z	*P*	OR (95%CI)
History of TB										
No					1.00 (Reference)					1.00 (Reference)
Yes	2.26	0.72	3.16	0.002	9.56 (2.35 ~ 38.82)	2.14	0.73	2.91	0.004	8.49 (2.01 ~ 35.81)
DRdiagnosis										
Normal					1.00 (Reference)					1.00 (Reference)
Abnormal	0.51	0.21	2.42	0.015	1.67 (1.10 ~ 2.54)	0.49	0.22	2.29	0.022	1.64 (1.07 ~ 2.50)
Smoke										
No					1.00 (Reference)					1.00 (Reference)
Yes	0.62	0.23	2.72	0.006	1.86 (1.19 ~ 2.90)	0.42	0.27	1.59	0.111	1.53 (0.91 ~ 2.58)
Sex										
Female					1.00 (Reference)					1.00 (Reference)
Male	0.52	0.21	2.44	0.015	1.68 (1.11 ~ 2.55)	0.31	0.25	1.25	0.210	1.37 (0.84 ~ 2.22)
Immunosuppressive diseases										
No					1.00 (Reference)					1.00 (Reference)
Yes	-1.40	0.60	-2.36	0.019	0.25 (0.08 ~ 0.79)	-1.34	0.60	-2.54	0.025	0.26 (0.08 ~ 0.84)

The following variables were included in the multivariate logistic regression model: History of TB, DR diagnosis, Smoke, Sex, and Immunosuppressive diseases

*Abbreviations: β* regression coefficient, *S*.*E *standard error, *Z *Z-statistic, *P *P-value, *OR *Odds Ratio, *CI* Confidence Interval

### Association between abnormal chest radiographs and TBI

In the primary analysis of the entire study population, we identified a significant association between abnormal chest radiographic findings and the risk of IGRA positivity. To further evaluate whether this association was independent, we constructed a series of multivariable logistic regression models (Table 3). In the initial Model 1, which was unadjusted for any potential confounders, abnormal chest radiographs were significantly associated with IGRA positivity, with an odds ratio (OR) of 1.67 (95% CI: 1.10–2.54). In Model 2, after adjusting for sex, based on known differences in immune response and TB susceptibility, the strength of this association remained largely unchanged (OR = 1.69, 95% CI: 1.05–2.51). Subsequently, in Model 3, we further included a history of immune diseases as a covariate, which was selected a priori based on biological plausibility and prior evidence of their association with TBI. The results demonstrated that abnormal chest radiographs remained a significant risk factor for IGRA positivity (OR = 1.66, 95% CI: 1.10–2.53). Finally, in the fully adjusted Model 4, which controlled for the most comprehensive set of confounders (sex, history of TB, smoking history, and history of immune diseases), the analysis confirmed that the association between abnormal chest radiographs and IGRA positivity remained stable and statistically significant (OR = 1.64, 95% CI: 1.08–2.49, *P* = 0.021).aModel1: Crude; bModel2: Adjust: Sex; cModel3: Adjust: Sex, Immunosuppressive diseases; dModel4: Adjust: Sex, History of TB, Smoke, Immunosuppressive diseases.

**Table 3:** Multivariable-adjusted models for the association between abnormal chest radiographs and igra positivity

Variables	Model1^a^		Model2^b^		Model3^c^		Model4^d^	
	OR (95%CI)	*P*	OR (95%CI)	*P*	OR (95%CI)	*P*	OR (95%CI)	*P*
DR diagnosis								
Normal	1.00 (Reference)		1.00 (Reference)		1.00 (Reference)		1.00 (Reference)	
Abnormal	1.67 (1.10–2.54)	0.015	1.69 (1.05–2.51)	0.018	1.66 (1.10–2.53)	0.017	1.64 (1.08–2.49)	0.021

*Abbreviations:* *OR *Odds Ratio, *CI *Confidence Interval

aModel1: Crude; bModel2: Adjust: Sex; cModel3: Adjust: Sex, Immunosuppressive diseases; dModel4: Adjust: Sex, History of TB, Smoke, Immunosuppressive diseases

**Fig. 1:**
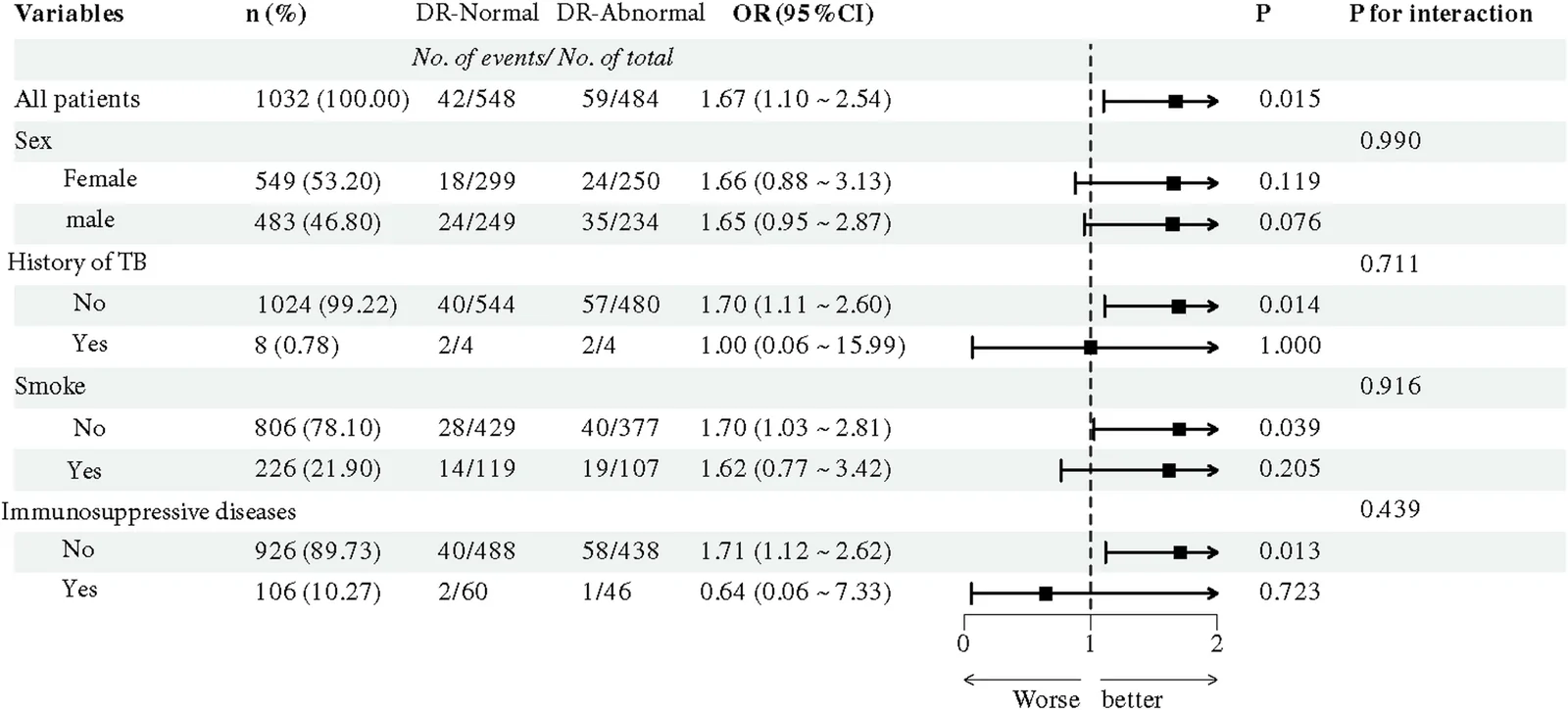
Subgroup analysis of the association between chest radiographic findings and IGRA results

We further conducted subgroup analyses to assess the association between abnormal chest radiographs and IGRA positivity across populations stratified by sex, history of tuberculosis, and history of immune diseases, and tested for potential interaction effects between these factors and abnormal chest radiographs (Fig. 1). The subgroup analyses indicated that the positive association between abnormal chest radiographs and IGRA positivity was consistent in direction across all subgroups, although the strength of the association and its statistical significance varied. The association did not reach statistical significance in analyses stratified by sex. However, it remained statistically significant in subgroups without a history of TB (OR = 1.70, 95% CI: 1.11–2.60, *P* = 0.014), without a smoking history (OR = 1.70, 95% CI: 1.03–2.81, *P* = 0.039), and without a history of immunosuppressive diseases (OR = 1.71, 95% CI: 1.12–2.62, *P* = 0.013). Formal tests for interaction, however, showed that neither sex (*P* for interaction = 0.990), history of tuberculosis (*P* for interaction = 0.711), smoking history (*P* for interaction = 0.916), nor history of immune diseases (*P* for interaction = 0.439) significantly modified the association between abnormal chest radiographic findings and IGRA positivity (all *P* for interaction > 0.05).

### Association between fasting blood glucose and IGRA positivity

We also analyzed the association between fasting blood glucose (FBG) levels and IGRA results, as summarized in Table 4. Blood glucose levels were 5.83 ± 1.44 mmol/L.in the IGRA-negative group and 5.47 ± 0.82 mmol/L in the IGRA-positive group, with the former being higher than the latter. In the total study population, 22.48% (232/1032) had abnormal FBG levels (> 6.1 mmol/L). Univariable analysis indicated a significant association between FBG status and IGRA positivity (χ² = 6.36, *P* = 0.012). Specifically, the proportion of individuals with abnormal FBG was significantly lower in the IGRA-positive group than in the IGRA-negative group (2.97% vs. 10.96%). To further investigate the influence of different glucose levels, we categorized FBG into three groups: normal (≤ 6.1 mmol/L), impaired fasting glucose (6.1–7.0 mmol/L), and diabetic range (> 7.0 mmol/L). Comparison among groups revealed a statistically significant difference in IGRA positivity rates across these FBG categories (χ² = 6.61, *P* = 0.037). Notably, the IGRA positivity rate was 10.38% (83/800) in the normal FBG group, 11.81% (15/127) in the impaired fasting glucose group, and lowest (2.86%, 3/105) in the diabetic-range FBG group (> 7.0 mmol/L).

**Table 4:** The association between blood glucose status and IGRA positivity

Variables	Total (*n* = 1032)	IGRA Negative (*n* = 931)	IGRA Positive (*n* = 101)	IGRA Positive Rates (%)	Statistic	*P*
FBG, Mean ± SD (mmol/L)	5.80 ± 1.40	5.83 ± 1.44	5.47 ± 0.82	-	t = 2.51	0.012
FBG Levels, n(%)					χ²=6.36	0.012
<=7.0 mmol/L	927	829 (89.04)	98 (97.03)	10.57		
>7.0 mmol/L	105	102 (10.96)	3 (2.97)	2.86		
FBG Levels, n(%)					χ²=6.61	0.037
<=6.1 mmol/L	800	717 (77.01)	83 (82.18)	10.38		
6.1- mmol/L	127	112 (12.03)	15 (14.85)	11.81		
>7.0 mmol/L	105	102 (10.96)	3 (2.97)	2.88		

*Abbreviations: t* t-test, *χ²* Chi-square test

**Fig. 2:**
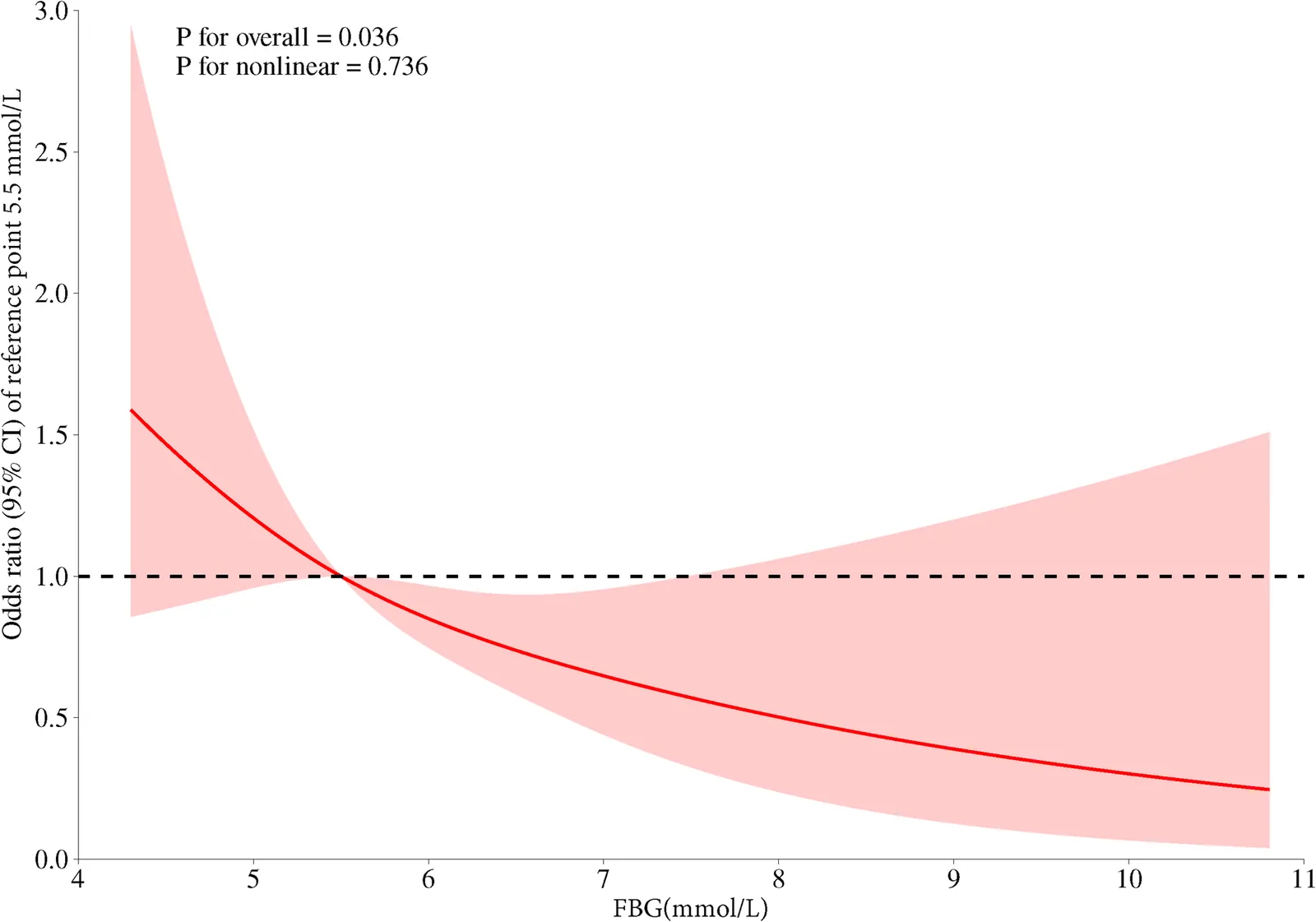
Association between fasting blood glucose and IGRA positivity using restricted cubic splines

A restricted cubic spline model was fitted to assess the relationship between fasting blood glucose and IGRA positivity, with adjustments for chest radiograph findings and history of TB. The reference value was set at 5.5 mmol/L (OR = 1.0), which corresponded to the median fasting blood glucose level in the study population. The overall association was statistically significant (*P* for overall = 0.036), while no significant nonlinearity was detected (*P* for nonlinear = 0.736). As shown in Fig. 2, the analysis demonstrated a significant inverse association between fasting blood glucose levels and IGRA positivity, with higher glucose levels corresponding to lower odds of a positive IGRA result throughout the observed range.

## Discussion

Huzhou is a low TB incidence area, with a reported TB incidence of 28.36 per 100,000 population in 2023 [14]. Through community-based IGRA screening of adults aged ≥ 65 years, we found an TBI prevalence of 9.79% (95% CI: 8.12%-11.75%), substantially lower than previous model-based estimates for global (23%) [2] and the national (38.36%) [23] levels. This discrepancy may accurately reflect the true infection pressure in areas with low TB transmission. Compared to high-incidence regions, limited transmission chains in low-incidence settings like Huzhou likely result in a lower cumulative infection rate among the population. Our real-world data help address the gap left by model-based estimates at the local level and offer direct evidence for accurately assessing regional TB transmission risk. Regarding generalizability, Huzhou shares key demographic and socioeconomic features with many other prefecture-level cities in eastern and southern China, including an aging population structure, low mobility, and well-established public health infrastructure. Our findings may therefore be cautiously generalizable to similar low-incidence settings, although they may not apply to high-incidence regions or younger, more mobile populations where transmission dynamics and risk factor profiles differ substantially. Further studies in diverse low-incidence settings are warranted to validate and extend these findings.

We found that a history of TB were significant risk factors for TBI, while immunosuppressive diseases were associated with lower IGRA positivity, which aligns with the existing body of literature [25]. This study did not find a significant association between age and TBI positivity. This may be due to the cross-sectional design of the study and the fact that all participants were aged 65 years or older, making it impossible to distinguish between recent and remote infections, which may have obscured any age effect to some extent. Notably, the number of female smokers in this study was too small, and the insufficient sample size of this group was one of the reasons why gender and smoking status showed no statistically significant differences in the multivariate analysis.

Through multivariable modeling and subgroup analyses, our study confirmed a significant and independent association between abnormal chest radiographic findings and IGRA positivity (adjusted OR = 1.67, 95% CI: 1.10–2.54). From a pathophysiological perspective, abnormalities on chest radiographs, such as fibrotic streaks, calcifications, or pleural thickening, are typically radiological evidence of previous tuberculosis infection that has healed or of old inflammatory lesions. The association between radiographic abnormalities and TBI was not statistically significant in some subgroups; however, interaction tests were all non-significant, and the risk estimates were consistent in direction across all subgroups (all ORs > 1). This suggests that the association between abnormal chest X-rays and TBI is consistent in direction across populations with different genders and risk behaviors. The variation in statistical significance across subgroups is more likely due to reduced statistical power from smaller sample sizes rather than true differences in effect. Our findings support the use of chest X-ray, a widely available and relatively inexpensive tool, as an effective initial screening method for identifying older adults at high risk of TBI.

A notable finding was the inverse association between elevated fasting blood glucose and IGRA positivity. This relationship, which was particularly evident at levels meeting the diabetes diagnostic threshold (FBG > 7.0 mmol/L), contradicts conventional risk expectations. Chronic hyperglycemia may alter immunometabolism, creating a microenvironment that compromises the survival and function of *M.tb*–specific T cells, thereby reducing IGRA reactivity. This immunocompromise of adaptive immunity is directly supported by the recent findings of Magodoro et al. [26], who demonstrated that while tuberculin skin test positivity was associated with dysglycemia, IGRA positivity showed no association, suggesting a selective impairment of the antigen-specific T-cell response measured by IGRA in the context of hyperglycemia. This is supported by studies [27–31] showing impaired Th1 and Th17 differentiation and decreased production of IFN-γ and IL-17 in TBI patients with diabetes—providing a mechanistic basis for potential false-negative IGRA results, as the assay relies fundamentally on IFN-γ release [32]. Given that these findings are specific to an elderly cohort, extrapolation requires caution. Further studies are needed to clarify the complex interaction between glucose metabolism, immune function, and TB infection in aging populations.

In summary, this large-scale community-based screening of older adults revealed a TBI prevalence of approximately 9.79% in a low TB incidence setting. Male sex and abnormal chest radiographs were identified as significant risk factors for TBI. These findings support prioritizing males and individuals with radiographic abnormalities for targeted TBI screening to enhance program efficiency. Additionally, elevated fasting blood glucose may mask TBI detection, indicating that the performance of IGRA in hyperglycemic populations requires further evaluation.

This study has several limitations. First, the cross-sectional design precludes causal inference regarding the association between risk factors and TBI. Future prospective studies incorporating longitudinal follow-up would help clarify the temporal relationships and the risk of progression from TBI to active TB. Second, as the study included only older adults from a low-incidence area, the generalizability of the findings to other populations should be approached with caution. Third, although multiple factors were collected and adjusted for in the models, residual confounding from unmeasured variables (such as socioeconomic status and occupational exposure history) may persist. Finally, TBI diagnosis relied solely on IGRA results. Although IGRA is widely recommended for TBI screening due to its high specificity, particularly in BCG-vaccinated populations, it is not a gold standard. Given that IGRA depends on T-cell-mediated immune responses, false-negative results may occur in immunocompromised individuals, including older adults with altered immunity, poorly controlled diabetes, or HIV infection. These conditions are relevant to our study population and may lead to an underestimation of TBI prevalence. False-positive results, though infrequent, may arise from infection with select non-tuberculous mycobacteria or laboratory variability.

Despite these limitations, we believe the findings provide valuable insights for TB control strategies in this population. This study concludes that in low-incidence areas, individuals with a prior TB history show significantly elevated IGRA positivity. The link between TBI and abnormal chest imaging suggests a practical role for radiography in screening. Furthermore, the inverse association of fasting blood glucose (FBG) with IGRA positivity introduces a new immunologic insight for aging populations that requires further exploration. Collectively, these findings deliver actionable local evidence to inform targeted screening strategies for the elderly.

## Supplementary Information

Supplementary Material 1.

## Data Availability

The data used in the manuscript is available upon request to the corresponding author.

## Abbreviations

β: Regression coefficient
S.E: Standard error
Z: Z-statistic
P: P-value
OR: Odds Ratio
CI: Confidence Interval
